# Inhibitory Effect and Potential Mechanism of *Lactobacillus plantarum* YE4 against Dipeptidyl Peptidase-4

**DOI:** 10.3390/foods11010080

**Published:** 2021-12-29

**Authors:** Jia Sha, Jiajia Song, Yechuan Huang, Yuhong Zhang, Hongwei Wang, Yu Zhang, Huayi Suo

**Affiliations:** 1 College of Food Science, Southwest University, Chongqing 400715, China; SghMjzSj@163.com (J.S.); jiajias@swu.edu.cn (J.S.); wanghw_1978@swu.com (H.W.); zhangyu_512@sina.cn (Y.Z.); 2 College of Bioengineering, Jingchu University of Technology, Jingmen 448000, China; huangyc@jcut.edu.cn; 3 Institute of Food Sciences and Technology, Tibet Academy of Agricultural and Animal Husbandry Sciences, Lhasa 850000, China; zhangyh75@126.com

**Keywords:** dipeptidyl peptidase-4, lactic acid bacteria, cell-free extracts, Caco-2 cells

## Abstract

This study investigated the inhibitory effect and mechanism of 12 LAB strains isolated from Chinese fermented foods on dipeptidyl peptidase-4 (DPP-4) using the Caco-2 cell model. The results showed that the inhibitory effect of cell-free extracts (CFEs) collected from each LAB strain on DPP-4 was higher than that of the cell-free excretory supernatants. The CFEs from *Lactobacillus plantarum* YE4 (YE4-CFE) exhibited the strongest DPP-4 inhibitory activity (24.33% inhibition). Furthermore, YE4-CFE altered the TNF and MAPK signaling pathways. Additionally, the YE4-CFE ultrafiltration fraction (<3 kDa) displayed a similar DPP-4 inhibitory activity to YE4-CFE. UHPLC-MS/MS identified 19 compounds with a relative proportion of more than 1% in the <3 kDa fraction, and adenine, acetylcholine, and L-phenylalanine were the top three substances in terms of proportion. Altogether, the inhibitory effect of YE4-CFE on DPP-4 was associated with the TNF and MAPK signaling pathways, and with the high proportion of adenine, acetylcholine, and L-phenylalanine.

## 1. Introduction

Diabetes is a metabolic disease whose major clinical symptom is high blood sugar levels or hyperglycemia [1]. The global consensus goal is to prevent the rise of diabetes and obesity by 2025. Type 2 diabetes mellitus (T2DM) is the most common type of diabetes, accounting for about 90–95% of all diabetes cases [2]. T2DM results from impaired insulin resistance and insulin secretion, and is primarily characterized by metabolic dysregulations that lead to a series of complications, such as diabetic retinopathy, nephropathy, cardiovascular disease, foot ulcers, and peripheral neuropathy [3]. Since these complications seriously affect a patient’s quality of life, research on the prevention and treatment of T2DM has become a popular research topic worldwide.

In recent years, various types of diabetes drugs have been developed to treat T2DM, such as sulfonylureas, thiazolidinediones, guanidines, α-glucosidase, and inhibitors. However, the major side effects of these drugs, including weight gain and hypoglycemia, prevent these treatments from achieving optimal blood glucose control for a long time [4]. Currently, dipeptidyl peptidase-4 (DPP-4) inhibitors are considered to have the greatest potential as anti-diabetic agents [5,6]. DPP-4 is a serine protease highly expressed on the lumen surface of intestinal epithelial cells. DPP-4 can cut and inactivate glucagon-like peptide-1 (GLP-1) and glucose-dependent insulinotropic polypeptide (GIP) at the postprandial stage, leading to the loss of their insulin-promoting activity. GLP-1 and GIP help to control the concentration of postprandial blood glucose [7,8]. DPP-4 inhibitors are small-molecule oral drugs for the treatment of T2DM. There are currently five types of DPP-4 inhibitors, namely vildagliptin, alogliptin, linagliptin, saxagliptin, and sitagliptin, which are all competitive and reversible inhibitors. When administered at therapeutic doses, an almost 90% inhibition rate of DPP-4 can be achieved within 15 min, and the inhibitory effect is maintained at approximately 70–90% after 24 h of administration [9]. However, synthetic DPP-4 inhibitors have some adverse effects, such as mild infection and headache [10]. Therefore, screening for DPP-4 inhibitors from natural sources has attracted considerable attention in recent years [6,11,12]. 

Lactic acid bacteria (LAB) are mostly derived from edible substances, which have the characteristics of being natural and safe. These characteristics have made their use in the rapid development of new agents for the prevention of diabetes a popular research area [13,14,15,16,17]. It has been reported that the cell-free excretory supernatants (CFSs) of *Lactobacillus plantarum* (*L. plantarum*) IF2-14 and ZF06-3 exert s significant inhibitory effect on DPP-4 in a concentration-dependent manner [6]. Heat-killed sonicated extracts of *L. plantarum* and *L. fermentum* also exhibit a DPP-4 inhibitory activity [12]. In addition, Tartary buckwheat and Chinese congee fermented by *L. paracasei* TK1501 are found to have an DPP-4 inhibitory activity [11,18]. However, the mechanism by which LAB inhibit DPP-4 activity has not been reported to date. 

In this study, the inhibitory effects of LAB strains on DPP-4 activity were evaluated in Caco-2 cells. We first identified LAB strains with a DPP-4 inhibitory activity based on the assays performed in Caco-2 cells. The mechanism of the LAB strain with the highest inhibitory activity against DPP-4 was further investigated by RNA-sequencing (RNA-seq) technology and quantitative real-time polymerase chain reaction (qRT-PCR) technology. Finally, the ultrafiltration technology and ultra-high-performance liquid chromatography-tandem-mass spectrometry (UHPLC-MS/MS) were used to identify the active components in the LAB strains with a DPP-4 inhibitory effect. To the best of our knowledge, this study identified, for the first time, the main active components and mechanism of a LAB strain with a DPP-4 inhibitory activity.

## 2. Materials and Methods

### 2.1. Materials and Reagents

Twelve LAB strains were isolated from Chinese Qula, yogurt, and preserved pickles from Chongqing (China) by our research team, and the specific strain information is shown in Table S1. The deMan Rogosa Sharpe (MRS) medium was purchased from Beijing Land Bridge Technology Inc. (Beijing, China). Dulbecco’s modified Eagle’s medium (DMEM; 4.5 g/L glucose) and 0.25% trypsin-EDTA solution were purchased from Gibco/Thermo Fisher Scientific Inc. (Waltham, MA, USA). Fetal bovine serum (FBS) was obtained from Biological Industries (Beit HaEmek, Israel). Phosphate-buffered saline (PBS), L-glutamine, agar, and penicillin-streptomycin liquid were supplied by Beijing Solarbio Technology Inc. (Beijing, China). Sitagliptin, Gly-Pro-PNA·HCl, was obtained from Sigma-Aldrich (St. Louis, MO, USA). RNase-Free water was purchased from Beyotime Biotechnology Inc. (Shanghai, China). Formic acid, methyl alcohol, ammonium acetate, TRIzol, and SYBR^®^Select Master Mix were produced from Thermo Fisher Scientific Inc. 

### 2.2. Caco-2 Cells Culture 

The caco-2 cells were purchased from the American Type Culture Collection (ATCC; Manassas, VA, USA). The Caco-2 cells were cultured in an incubator at 37 °C, with a 5% CO_2_ atmosphere, in DMEM containing 10% FBS, 1% penicillin-streptomycin liquid, and L-glutamine at a concentration of 2 mM. When the cells reached a confluence of about 80%, they were digested with 0.25% trypsin-EDTA and were passaged. The medium was replenished with fresh medium every other day during the first week and every day after the first week. Caco-2 cells from passage 6 to 45 were used in this study.

### 2.3. Determination of DPP-4 Activity in Caco-2 Cells

Caco-2 cells were seeded into 96-well plates at a low density (1 × 10^5^ cells/mL) and high density (7 × 10^5^ cells/mL), respectively. The DPP-4 activity in the cells was measured on the 2nd, 4th, 7th, 10th, 14th, 18th, and 21st days after seeding. For determination of the DPP-4 activity, after washing the cells in each well with PBS, 100 µL of Gly-Pro-PNA·HCl at a concentration of 4 mM was added and the plate was incubated at 37 °C for 60 min. Afterwards, the absorbance (Abs) of the reacted samples was measured at 405 nm in a SYNERGY H1 microplate reader (BioTek, Winooski, VT, USA). 

In order to determine the culture time of Caco-2 cells, the inhibitory effect of sitagliptin on DPP-4 was determined. The effect of sitagliptin on the DPP-4 activity was assayed using a previously reported method [19]. The Caco-2 cells were seeded into 96-well plates at a density of 7 × 10^5^ cells/mL, and the DPP-4 activity was measured on the 2nd, 4th, 7th, and 10th days after seeding. Before determination of the DPP-4 activity, the cells in each well were washed with PBS, and 100 µL sitagliptin in PBS at concentrations of 10^−4^, 10^−3.5^, 10^−3^, 10^−2^, and 10^−1^ mM were added separately, and the plate was incubated at 37 °C for 30 min. The PBS solution was regarded as the positive control. The sample control and negative control were the same as the sample group and positive group, respectively, except that no cells were seeded. Then, the cells in each well were washed with PBS to completely remove the treatment medium. Subsequently, 100 µL Gly-Pro-PNA∙HCl at a concentration of 4 mM was added to each well and was incubated at 37 °C for 60 min. Afterwards, the Abs of the reacted samples was measured at 405 nm in a SYNERGY H1 microplate reader (BioTek). The inhibition rate of DPP-4 (DIR) was calculated using the following Formula (1).

(1) $$\mathrm{DIR}\left(\%\right)=1-\frac{{\mathrm{Abs}}_{\mathrm{sample}}-{\mathrm{Abs}}_{\mathrm{sample}\;\mathrm{control}}}{{\mathrm{Abs}}_{\mathrm{positive}\;\mathrm{control}}-{\mathrm{Abs}}_{\mathrm{negative}\;\mathrm{control}}}\times 100$$

### 2.4. Preparation of Cell-Free Excretory Supernatant (CFS) and Cell-Free Intracellular Extract (CFE)

The preparation of CFS was performed using a previously described procedure [6], with some modifications. The LAB strains were cultured at 37 °C for 18 h, and the culture was centrifuged at 4000× *g* for 10 min to collect the cell pellets. The cell pellets were washed twice with PBS solution and adjusted to 1 × 10^8^ CFU/mL. The suspended cell pellets were inoculated in the MRS medium at 2% (*v*/*v*) and then were cultured at 37 °C for 12 h. Subsequently, the culture was centrifuged at 4000× *g* for 10 min and the supernatant obtained was designated as CFS. After that, the CFS was neutralized to pH 7.4 and was lyophilized. 

The CFE of the LAB was prepared according to a method described in a reported study [6], with some modifications. The LAB strains were cultured at 37 °C for 18 h and the culture was centrifuged at 4000× *g* for 10 min to collect the cell pellets. The cell pellets were washed twice with PBS solution and adjusted to 1 × 10^8^ CFU/mL. The cell pellets were subjected to ultrasonic fragmentation in an ice bath under the following conditions: power at 30% for 3 s, stopped for 5 s, and sustained for 15 min. Finally, the solution was centrifuged at 4 °C, 12,000× *g* for 10 min, and the supernatant was neutralized to pH 7.4 before lyophilization.

### 2.5. Determination of the Inhibitory Effects of CFS and CFE on DPP-4 Activity

Caco-2 cells were seeded into 96-well plates at a density of 7 × 10^5^ cells/mL. The DPP-4 activity was measured on the fourth day after seeding. Before the determination of the DPP-4 activity, the Caco-2 cells were washed with PBS. Then, 100 μL of CFS, CFE, or their vehicles, filtered through a 0.22 μm membrane, were added to the corresponding wells and the plate with Caco-2 cells was pre-incubated for 12 h. DMEM containing 10% FBS and 2 mM L-glutamine was used as CFE’s vehicle, while MRS in DMEM containing 10% FBS and 2 mM L-glutamine was used as CFS’s vehicle. The blank group was the same as the vehicle group, except that no cells were seeded. After that, 100 µL Gly-Pro-PNA∙HCl at a concentration of 4 mM was added into each well and the plate was incubated at 37 °C for 60 min. Afterwards, the Abs of the reacted samples was measured at 405 nm in a SYNERGY H1 microplate reader (BioTek). The DIR was calculated using the following formula (2).

(2) $$\mathrm{DIR}\left(\%\right)=1-\frac{{\mathrm{Abs}}_{\mathrm{sample}}-{\mathrm{Abs}}_{\mathrm{blank}}}{{\mathrm{Abs}}_{\mathrm{vehicle}}-{\mathrm{Abs}}_{\mathrm{blank}}}\times 100$$

### 2.6. qRT-PCR Analysis

The total RNA from the Caco-2 cells was extracted using TRIzol Reagent. The purity and concentration of the RNA were measured on a P100+ micro-ultraviolet spectrophotometer (Pultton, Ann Arbor, MI, USA). The total RNA was reverse transcribed into cDNA using the First Strand cDNA Synthesis Kit (Thermo Fisher Scientific Inc.), and was then amplified in a 20-μL PCR system. The qPCR reaction was performed on a CFX Connect System (Bio-Rad Laboratories, Hercules, CA, USA). PPIA and GAPDH were selected as the internal control genes for the DPP-4 gene and other genes, respectively. The sequences of the primers used are listed in Table S2. The relative mRNA expression was calculated through the 2^−^^△△Ct^ method.

### 2.7. RNA-Seq Analysis

Caco-2 cells were seeded into six-well plates at a density of 7 × 10^5^ cells/mL. The medium was replenished with fresh medium every other day. On the fourth day after seeding, the Caco-2 cells were washed with PBS and then treated with 10 mg/mL of sample for 12 h. Subsequently, the cells were washed with PBS to remove the treatment medium and 1 mL of TRIzol reagent was added to each well, and the cell lysates were repeatedly pipetted up and down to make TRIzol fully in contact with the cells. Afterwards, the samples were quickly frozen with liquid nitrogen. Finally, the RNA-seq analysis of the samples was performed using a previously described procedure [20,21].

### 2.8. Analysis of Active Compounds of CFE from LAB Strain Inhibiting DPP-4

The samples with a DPP-4 inhibitory activity were fractionated using ultrafiltration tubes (Millipore Corporation, Bedford, MA, USA) with molecular mass cutoffs of 3, 5, 10, and 30 kDa. A 30-kDa ultrafiltration tube was first used to divide the sample into >30 kDa and <30 kDa, and then a 10 kDa ultrafiltration tube was used to divide the <30 kDa sample into 10–30 kDa and <10 kDa. Similarly, 5 kDa and 3 kDa ultrafiltration tubes were successively used for additional gradual separation. Finally, the collected fractions were lyophilized and preserved at −80 °C. The <3 kDa fractions of CFE from LAB strain inhibiting DPP-4 were further analyzed by UHPLC-MS/MS using a method described in a previous study [22].

### 2.9. Statistical Analysis

All of the tests were performed at least in triplicate. The results are expressed as the mean ± standard deviation. All data were analyzed using one-way ANOVA with Tukey′s test to assess significant differences between means (*p* < 0.05) using the IBM SPSS 22 (IBM Corporation, Armonk, NY, USA).

## 3. Results

### 3.1. Characterization of DPP-4 Activity in Caco-2 Cells

**Figure 1:**
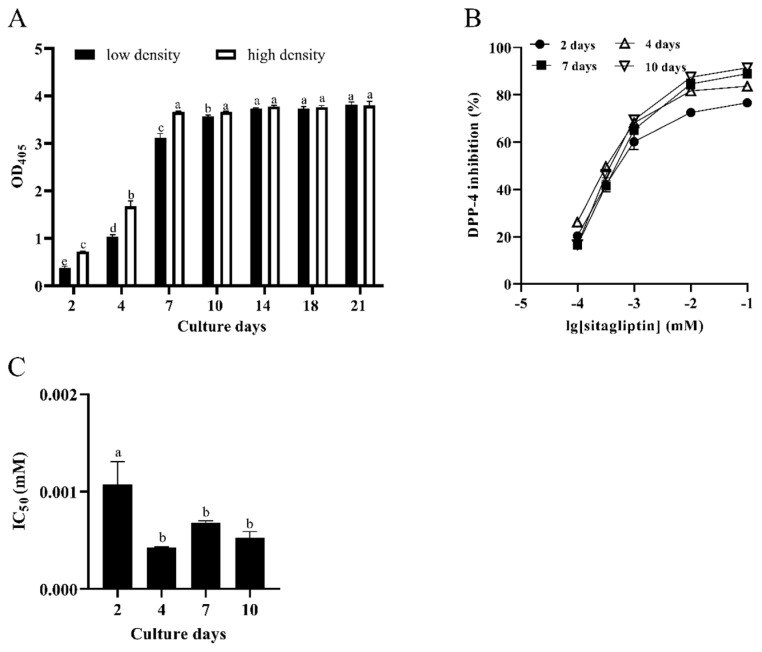
Characterization of DPP-4 activity in Caco-2 cells. (**A**) Effects of culture time and seeding density on the DPP-4 activity in Caco-2 cells. Different letters (a–e) on the column with the same color indicate significant differences (*p* < 0.05). (**B**) Effects of sitagliptin on DPP-4 activity in Caco-2 cells. (**C**) IC_50_ value of sitagliptin inhibiting DPP-4 at different culture days. Different letters (a,b) indicate significant differences (*p* < 0.05). Each value represents the mean ± standard deviation (*n* = 3). Note, low density, 1 × 10^5^ cells/mL; high density, 7 × 10^5^ cells/mL.

As shown in Figure 1A, the activity of DPP-4 in Caco-2 cells seeded at a low density (1 × 10^5^ cells/mL) increased with the increase of the culture time. On the 14th day after seeding, the DPP-4 activity in Caco-2 cells stopped increasing and reached a plateau phase (*p* < 0.05). Likewise, the activity trend of DPP-4 in Caco-2 cells seeded at a high density (7 × 10^5^ cells/mL) was similar to that at a low density. The difference was that the DPP-4 activity of Caco-2 cells seeded at a high density reached a plateau phase after 7 days from seeding (*p* < 0.05). These results demonstrated that the activity of DPP-4 reaches a plateau phase faster in the Caco-2 cells seeded at a higher density.

Sitagliptin, a recognized DPP-4 inhibitor, was used as a reference compound to verify the DPP-4 activity. Since, in this study, the DPP-4 activity in Caco-2 cells seeded at a high density reached the plateau phase faster, the Caco-2 cells seeded at a high density were used to investigate the effect of sitagliptin on culture time, so as to determine the optimal culture time of the cell model. As shown in Figure 1B, the results revealed that the DIR also gradually increased with the increase of the concentration of sitagliptin, indicating a dose-dependent relationship. The half inhibitory concentration (IC_50_) values of sitagliptin on the 2nd, 4th, 7th, and 10th days after seeding were 1.056, 0.426, 0.680, and 0.524 μM, respectively (Figure 1C). In comparison with the 7th days and 10th days, there was no notable difference in the IC_50_ value of sitagliptin on the 4th day (*p* < 0.05). This finding demonstrated that the features of DPP-4 in Caco-2 cells seeded at a high density (7 × 10^5^ cells/mL) had no obvious change (*p* < 0.05) after 4 days from seeding. In other words, Caco-2 cells seeded at a high density could be used for screening LAB strains after 4 days from seeding.

### 3.2. Effects of LAB Strains on DPP-4 Activity

**Figure 2:**
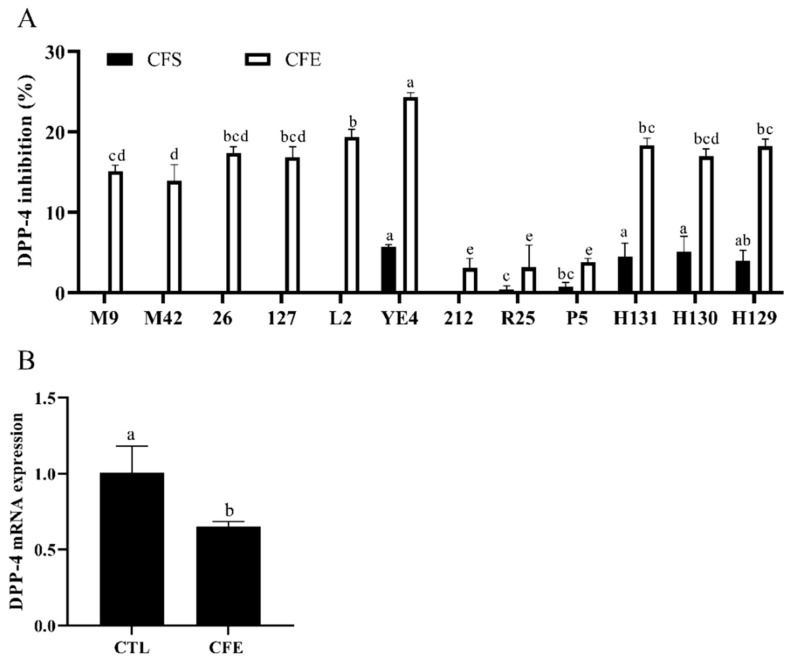
(**A**) Effects of different lactic acid bacteria (LAB) strains on the DPP-4 inhibitory activity in Caco-2 cells. CFS, cell-free excretory supernatants; CFE, cell-free extracts. (*p* < 0.05). (**B**) Effects of YE4-CFE on the mRNA expression of DPP-4 in Caco-2 cells. CFE, treatment group after YE4-CFE interacted with Caco-2 cells; CTL, control group. Each value represents the mean ± standard deviation (*n* = 3). Different letters (a–e) on the column with the same color indicate significant differences (*p* < 0.05).

The CFSs and CFEs were prepared from 12 strains of LAB isolated from Chinese fermented foods to determine their inhibitory effects on the DPP-4 activity in Caco-2 cells. The results shown in Figure 2A reveal that the DIR of the CFSs ranged from 0 to 5.68%. However, the CFEs of all LAB strains showed an inhibitory activity against DPP-4, ranging from 3.09 to 24.33%. Notably, the CFE of the YE4 strain had the highest DIR of 24.33%, which indicated that it is a potential strain with a DPP-4 inhibitory activity. We further characterized the YE4 strain. As shown in Figure S1, the colony morphology of the YE4 strain was uniform, and the shape was round. The colony of the YE4 strain was purple in color and appeared as a short-rod after Gram staining, indicating that the bacterial strain was Gram-positive. Homology alignment and phylogenetic tree construction based on the 16S rDNA sequences of the YE4 strain revealed 100% similarity with *L. plantarum* F1031, and its registration number was MW719476.1. Figure S1E shows that strain YE4 can utilize 22 carbohydrates, and was identified as *L. plantarum* by the API 50CH lab plus system. These results revealed that the strain YE4 is identified as *L. plantarum,* which is abbreviated as *L. plantarum* YE4. The CFE of YE4 was designated as YE4-CFE.

The mRNA expression of DPP-4 was analyzed to determine whether YE4-CFE affected the activity of DPP-4 by regulating the expression of DPP-4. The results revealed that the mRNA expression of DPP-4 was dramatically reduced after treatment with YE4-CFE (*p* < 0.05; Figure 2B). The data shown in Figure 2 also indicate that YE4-CFE not only suppressed the DPP-4 activity, but also the mRNA expression of DPP-4 in Caco-2 cells.

### 3.3. Overview of the Transcriptional Changes

To understand the molecular regulation of DPP-4 activity by YE4-CFE in Caco-2 cells, we performed RNA-seq analysis of Caco-2 cells treated with YE4-CFE. As shown in Table S3, each sample generated more than 6G raw reads and the Q20 and Q30 were higher than 97 and 93%, respectively, indicating the high throughput and quality of the RNA-Seq data.

**Figure 3:**
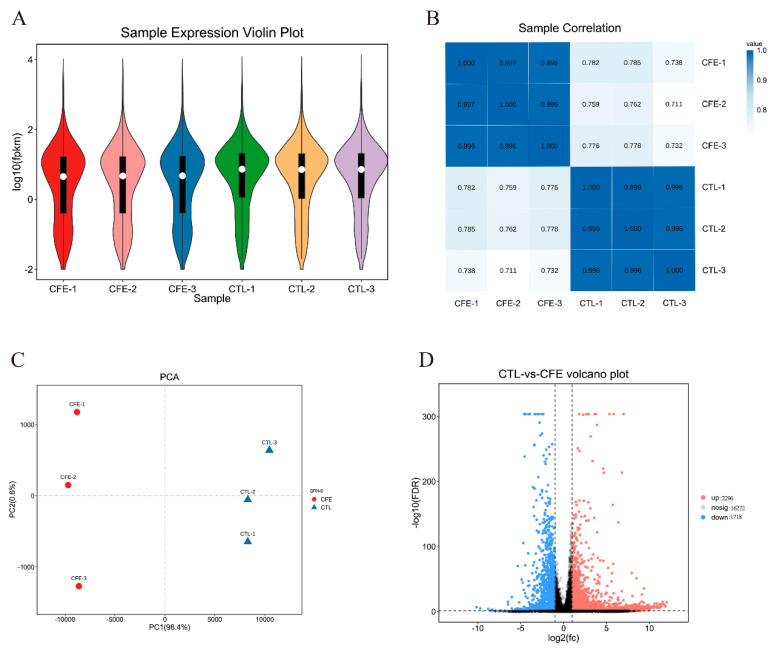
Effects of the cell−free extracts (CFEs) collected from *L. plantarum* YE4 (YE4−CFE) on the global gene expression profiling in Caco−2 cells. (**A**) Violin plot. The white dot in the plot represents the median, and the black rectangle is the range from the lower quartile to the upper quartile. The black line running through the violin represents the minimum non−abnormal value to the maximum non-abnormal value. (**B**) Sample correlation heat map. The value in each small box is the Pearson correlation coefficient (R). (**C**) Principal component analysis (PCA). PC1 represents the first principal component and PC2 represents the second principal component. (**D**) Volcano map. The red dots indicate the upregulated differentially expressed genes (DEGs). The blue dots indicate the downregulated DEGs. However, there is no significant difference in the expression of the genes in black. DEGs were estimated by FDR < 0.05 and |log2(FC)| > 1. CFE, treatment group after YE4−CFE interacted with Caco−2 cells; CTL, control group.

The value of the FPKM (fragments per kilobase of transcript per million mapped reads) can be used to reflect the gene expression level. The violin map based on the FPKM value of each gene is commonly used for the visualization of the gene abundance expression. As shown in Figure 3A, the comparison of the gene abundance between the control (CTL) group and CFE group revealed a prominent distinction. Pearson correlation coefficient R was used to evaluate the sample correlation, so as to evaluate the reliability of the experimental results and the stability of the operation. It was found, as shown in Figure 3B, that there were high intra-group correlations in the CFE group and CTL group, whereas the correlation between the CTL group and CFE group was low, indicating that the repeatability of the intra-group repeated samples was good. Principal component analysis (PCA) is commonly used as a multivariate statistical analysis method to examine the distribution between samples. The higher the sample similarity, the closer the distribution. The first principal component (PC1) and second principal component (PC2) contributed 99.2% of the total, and the CFE and CTL groups were distributed in different regions (Figure 3C). These data further confirmed that YE4-CFE has a significant effect on Caco-2 cells.

The volcano map can intuitively reflect the distribution of the entire set of differentially expressed genes (DEGs), and explain both the multiple and the significant level of DEGs. The number of DEGs observed under YE4-CFE treatment is shown in Figure 3D. There were 16,272 genes that exhibited no significant differential expression between the CFE group and the CTL group. However, compared with the CTL group, 2296 genes were markedly upregulated and 1718 genes were markedly downregulated in the CFE group. Based on the above findings, it can be concluded that the gene expression profile of cells was noticeably changed after Caco-2 cells interact with YE4-CFE.

### 3.4. GO Enrichment and KEGG Enrichment Analysis of DEGs

**Figure 4:**
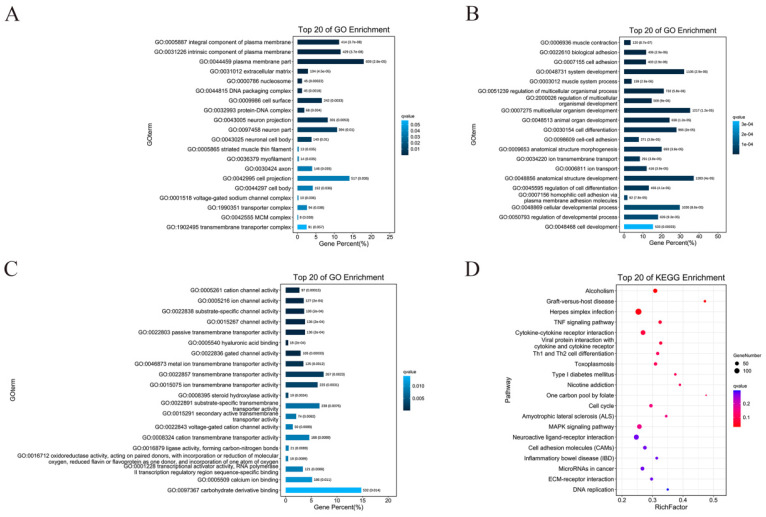
Effects of the cell-free extracts (CFEs) collected from *L. plantarum* YE4 (YE4-CFE) on GO enrichment and KEGG enrichment analysis. (**A**–**C**) GO enrichment analysis. (**A**) Cell components. (**B**) Biological process. (**C**) Molecular functions. The horizontal axis represents the percentage of the GO term number to the number of differentially expressed genes (DEGs). The vertical axis represents the GO term information. (**D**) KEGG enrichment analysis. The horizontal axis indicates the rich factor (RF). The vertical axis indicates the pathway. The size of the dots in the pathway represents the quantity of DEGs, and the color of dots denote the different range of q-values. RF refers to the ratio of DEGs enriched in this pathway to the genes annotated to this pathway. The q value is the corrected *p* value after multiple hypothesis tests. The q value is in the range of 0–1, which, if it is close to 0, indicates more significant enrichment. CFE, treatment group after YE4-CFE interacted with Caco-2 cells; CTL, control group.

Gene ontology (GO) is known as a standardized gene function category database that provides a system for hierarchically classifying genes or their products into terms, organized in a graph structure, in any organism [23]. In this study, we observed 60 significantly enriched subcategories in DEGs to the GO databases (FDR < 0.05). Among the DEGs in the CTL and CFE groups, the significant functional differences in the cell component (CC) enrichment were as follows: integral component of the plasma membrane, intrinsic component of the plasma membrane, the plasma membrane part, etc. (Figure 4A). The marked functional differences in biological process (BP) enrichment were the following: muscle contraction, biological adhesion, cell adhesion, etc. (Figure 4B). In addition, the prominent functional differences in molecular functions (MF) enrichment were as follows: cation channel activity, ion channel activity, substrate-specific channel activity, etc. (Figure 4C).

In biology, different genes coordinate and perform their own biological functions. Pathway analysis is the embodiment of the gene biological functions. KEGG is a major database of pathways, which provides all relevant metabolic pathways, such as amino acids and carbohydrates in organisms [24]. The results of the enrichment analyses, shown in Figure 4D, revealed that DEGs were primarily annotated in alcoholism, graft-versus-host disease, herpes simplex infection, TNF signaling pathway, cytokine and cytokine receptor, viral protein interaction with cytokine and cytokine receptor, Th1 and Th2 cell differentiation, toxoplasmosis, and type 1 diabetes (FDR < 0.05). The main uppermost number of DEGs enrichment in the KEGG pathways were displayed in herpes simplex infection, neuroactive ligand-receptor interaction, cytokine−cytokine receptor interaction, and MAPK signaling pathway. The results revealed that YE4-CFE was most likely to affect the activity of DPP-4 in Caco-2 cells through the above pathway.

### 3.5. Effect of YE4-CFE on the Expression of TNF Signaling Pathway-Related Genes

**Figure 5:**
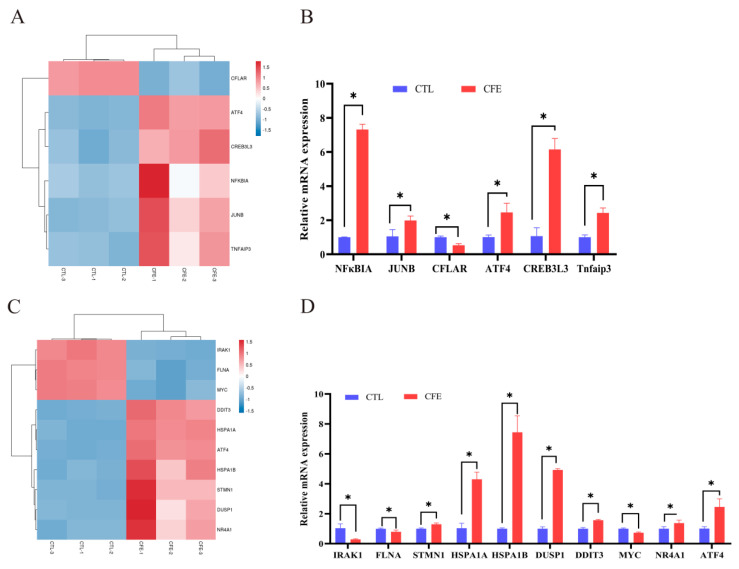
Effects of the cell−free extracts (CFEs) collected from *L. plantarum* YE4 (YE4−CFE) on the genes related to the TNF and MAPK signaling pathway. (**A**) Heat map of the TNF signaling pathway. (**B**) Effect of YE4−CFE on the expression of the TNF pathway−related genes in Caco−2 cells. (**C**) Heat map of the MAPK signaling pathway. (**D**) Effect of YE4−CFE on the expression of the MAPK pathway−related genes in Caco−2 cells. CFE, treatment group after YE4−CFE interacted with Caco−2 cells; CTL, control group. Asterisks denote significant difference (* *p* < 0.05) between the CTL group and CFE group.

According to the results of the RNA-seq analysis shown in Figure 5A, compared with the CTL group, the CFLAR gene of the TNF signaling pathway was markedly downregulated, while other TNF signaling pathway genes, namely ATF4, CREB3L3, NFκBIA, JUNB, and Tnfaip3, were notably upregulated (*p* < 0.05). The mRNA expression of the same genes above was measured by qRT-PCR analysis. As shown in Figure 5B, the results revealed that the upward and downward trends of these genes were consistent with the RNA-Seq analysis results. The mRNA expression of NFκBIA was increased the most, by 7.32-fold, followed by those of CREB3L3, ATF4, Tnfaip3, and JUNB, by 6.1-, 2.46-, 2.43-, and 1.98-fold respectively, while the mRNA expression of CFLAR was downregulated by 0.53-fold relative to that in the CTL group (*p* < 0.05).

### 3.6. Effect of YE4-CFE on the Expression of MAPK Signaling Pathway-Related Genes

The RNA-seq analysis results (Figure 5C) revealed that the expression of the MAPK signaling pathway-related genes IRAK1, FLNA, and MYC were dramatically downregulated, while other MAPK signaling pathway-related genes, namely DDIT3, HSPA1A, ATF4, HSPA1B, STMN1, DUSP1, and NR4A1, were markedly upregulated compared with the CTL group (*p* < 0.05). The results in Figure 5D show that the mRNA expression of the HSPA1B gene was increased the most, by 7.44-fold, followed by those of DUSP1 (4.93-fold), HSPA1A (4.30-fold), ATF4 (2.46-fold), DDIT3 (1.57-fold), NR4A1 (1.37-fold), and STMN1 (1.30-fold) compared with those in the CTL group (*p* < 0.05). After the Caco-2 cells were treated with YE4-CFE, the mRNA expression of the IRAK1, MYC, and FLNA genes decreased 0.29-, 0.73-, and 0.80-fold, respectively (*p* < 0.05). These data demonstrate that the upregulation and downregulation of these genes are similar to those obtained by the RNA-seq analysis.

### 3.7. Analysis of the Effects of the Active Components of YE4-CFE against DPP-4 Activity

**Figure 6:**
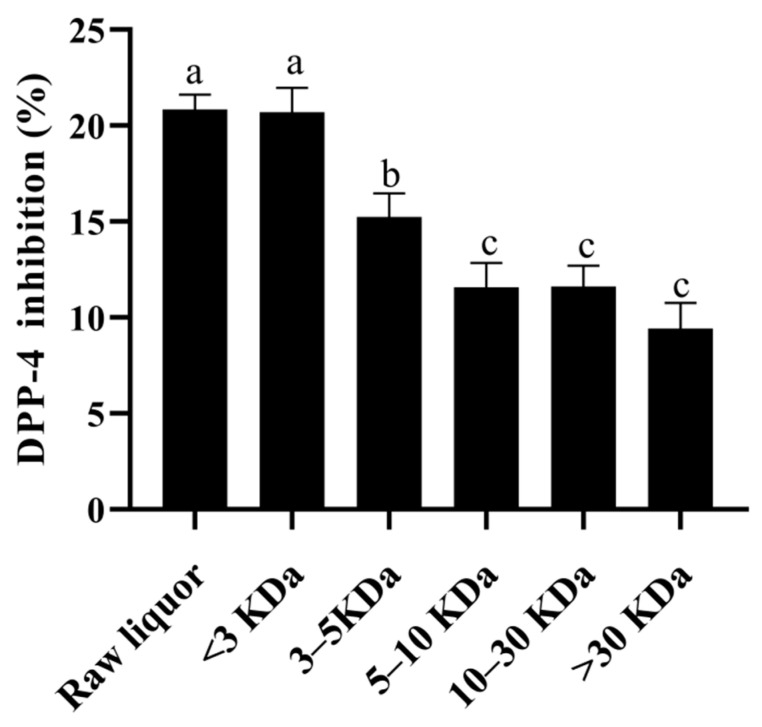
Effects of the cell-free extracts (CFEs) collected from *L. plantarum* YE4 (YE4-CFE) with different molecular weight components on the inhibitory activity of DPP-4. CFEs, cell-free extracts; YE4-CFE, the CFE of *L. plantarum* YE4. Each value represents the mean ± standard deviation (*n* = 3). Different letters (a–c) indicate significant differences (*p* < 0.05).

Ultrafiltration can fractionate the sample into different molecular weight components by using different retention values, so as to separate the target material from other molecular weight impurities. According to the results in Figure 6, the greater the molecular weight of the fractions from YE4-CFE, the lower its DIR in Caco-2 cells. When the molecular weight of the YE4-CFE component was less than 3 kDa, the DIR was the highest (20.70%), followed by the 3–5 kDa components and 5–10 kDa components with DIR of 15.23 and 11.56%, respectively. The component with a molecular weight greater than 30 kDa had the weakest DIR, of 9.44%. The data obtained in this study clearly indicate that YE4-CFE exerted an inhibitory effect on DPP-4 in Caco-2 cells, mainly through small molecules less than 3 kDa.

UHPLC-MS/MS was used to identify substances with a molecular weight of less than 3 kDa in YE4-CFE. The results revealed that there were 19 compounds with a relative proportion of more than 1% (Table 1). Most of these compounds were categorized as imidazopyrimidines, carboxylic acids and derivatives, and acetylcholine, comprising 16.991, 14.361, and 7.479% YE4-CFE, respectively. It was further found that adenine is present at the highest proportion in YE4-CFE (10.573%), followed by acetylcholine (7.479%) and L-phenylalanine (7.177%).

## 4. Discussion

In recent years, DPP-4 inhibitors have been considered as a powerful treatment for patients with T2DM [4]. Due to their natural properties, LAB have become a new source to screen for DPP-4 inhibitors [6,12]. A previous study has reported that 21 strains showed a DPP-4 inhibitory activity on porcine DPP-4, and the DIR of the CFE samples ranged from 8.0 to 33.3% [6]. In addition, 15 heat-killed sonicated extracts of various LAB strains from human infant feces had the ability to inhibit DPP-4, with inhibition rates ranging from 10 to 25% [12]. However, to date, only human recombinant DPP-4 or DPP-4 from porcine have been used for screening LAB strains by in vitro chemical methods. Compared with in vitro chemical methods, experimental cellular assays are more conducive to evaluating the activity and expression of DPP-4 in an in vitro environment. Accordingly, in order to better characterize the activity of DPP-4, this study also used an established Caco-2 cell model to screen LAB against DPP-4 activity. It is worth mentioning that the enzyme activity of DPP-4 in Caco-2 cells seeded at a high density (7 × 10^5^ cells/mL) reached a plateau phase faster than that seeded at a low density. We further found that the IC_50_ value of sitagliptin to DPP-4 had no obvious disparity when Caco-2 cells were seeded at a high density and tested at 4 days from seeding. Accordingly, in this study, Caco-2 cells seeded at a high density cultured for 4 days after seeding were used to screen for LAB with activity against DPP-4. The results indicated that the CFE of all LAB strains had an inhibitory effect on DPP-4 (3.09–24.33%), but CFS exhibited a low inhibitory activity against DPP-4 (0–5.68%). It is important to mention that YE4-CFE had the highest rate of inhibition of DPP-4, reaching 24.33%. In addition, it is also important to point out that the DPP-4 activity was correlated to the gene expression of DPP-4. As early as 1991, Yoshio found that with the increase of the Caco-2 cell culture time, the mRNA expression and activity of DPP-4 also increased [25]. The treatment of Caco-2 cells with grape seed-derived procyanidins caused a decrease in the DPP-4 activity and gene expression [26]. In the present study, after treatment of Caco-2 cells with YE4-CFE, the mRNA expression of DPP-4 significantly decreased (*p* < 0.05). To the best of our knowledge, this research study is the first to use Caco-2 cells to evaluate the inhibitory effect of LAB strains on DPP-4 activity, and found that YE4-CFE not only decreased the activity of DPP-4, but also suppressed the mRNA expression of DPP-4 in these cells.

RNA-seq analysis is a general term for high-throughput sequencing analysis of various types of transcription samples, and this technology has rapidly advanced in recent years [27]. It can explore the function and structure of genes at the overall level and reveal the molecular mechanism of specific biological processes [28]. Therefore, this study used RNA-seq to comprehensively screen changes in gene expression in Caco-2 cells after treatment with YE4-CFE. The violin plot and correlation heat map showed that the gene distribution of the CFE group and CTL group was different. The CFE group and the CTL group were distributed in different regions in the PCA plot. It was further found that there were 4014 DEGs between the CFE and CTL groups, of which 2296 were upregulated and 1718 were downregulated. In addition, the GO enrichment analysis revealed that these DEGs were mainly related to the integral component of the plasma membrane, biological adhesion, cell adhesion, and cation channel activity. In addition, the KEGG pathway enrichment analysis revealed that these DEGs were annotated in the TNF, MAPK, and other signaling pathways. TNF is a pro-inflammatory cytokine secreted by visceral adipose tissue [29]. The elevated TNF levels were previously found to be relevant to insulin resistance and T2DM [30]. *L. plantarum* MTCC5690 and *L. fermentum* MTCC5689 were shown to alleviate insulin resistance in mice by reducing the expression of TNF and other genes, thereby preventing the development of diabetes [31]. The non-specific inhibitor of TNF, pentoxifylline, was reported to reduce the transcriptional activity of DPP-4 in a streptozotocin-induced diabetic rat [32]. The MAPK signaling pathway is a very important inflammatory signaling pathway [33]. It has been reported that if systemic chronic inflammation is not treated in time, it could lead to diseases such as T2DM [34]. Previous research has also suggested that the MAPK signaling pathway is upregulated by soluble DPP-4 [35]. The clerodane diterpene 16-hydroxycleroda-3,13-dien-15,16-olide (HCD) derived from *Polyalthia longifolia* inhibited ERK phosphorylation in the MAPK signaling pathway, and also inhibited the activity of soluble DPP-4 [36]. As expected, our results revealed that the six genes related to the TNF signaling pathway showed a similar trend as that revealed by the RNA-seq analysis results, including the significant downregulation of the mRNA expression of CFLAR, and upregulation of the mRNA expression of ATF4, CREB3L3, NFκBIA, JUNB, and Tnfaip3. Likewise, the 10 genes related to the MAPK signaling pathway also showed similar changes in the RNA-seq analysis. After Caco-2 cells were treated with YE4-CFE, the mRNA expression of IRAK1, FLNA, and MYC was remarkably relieved, while the mRNA expression of DDIT3, HSPA1A, ATF4, HSPA1B, STMN1, DUSP1, and NR4A1 was significantly increased (*p* < 0.05). Overall, this study demonstrated that the inhibitory effect of YE4-CFE on DPP-4 was relevant to its regulation of gene expression in the TNF and MAPK signaling pathways.

Ultrafiltration is a very common and convenient method for separating bioactive substances. The substances with a molecular weight of 1–1.25 kDa separated by ultrafiltration exhibited the highest inhibition rate on DPP-4 after hydrolysis of porcine skin gelatin [37]. Ultrafiltration fractions of <1.5 kDa obtained from halibut skin gelatin hydrolysate (HSGH) and tilapia skin gelatin hydrolysate (TSGH) were also found to have the strongest DPP-4 inhibitory activity [38]. Similarly, in this study, it is worth noting that different molecular weight components from YE4-CFE exhibited great differences in DIR in Caco-2 cells. The component with highest DIR was the <3 kDa (20.70%), which was not much different from the raw liquor (20.84%) (*p* < 0.05). Besides that, the DIR decreased with the increasing molecular weight. These findings indicated that the active components of YE4-CFE with a DPP-4 inhibitory effect were mainly small molecules of less than 3 kDa. We further analyzed these substances by UHPLC-MS/MS. There were 19 compounds with a relative proportion of more than 1%, and their relative proportion reached 69.59% in total. Among them, adenine, acetylcholine, L-phenylalanine, guanine, 2-hydroxycinnamic acid, indole-3-acrylic acid, uracil, and indole-3-lactic acid have been reported to exist in the CFS of *L. plantarum* BLP12, which was shown to markedly restrain the activity of DPP-4 [39]. Thus, it could be inferred that the 19 compounds of less than 3 KDa in YE4-CFE, particularly the high percentage of adenine (10.573%), acetylcholine (7.479%), L-phenylalanine (7.177%), and guanine (6.418%), were responsible for the inhibitory effect on DPP-4.

## 5. Conclusions

In summary, in this study, we isolated a LAB strain from Chinese Qula, namely *L. plantarum* YE4. The YE4-CFE notably suppressed the activity and the mRNA expression of DPP-4 in Caco-2 cells. The inhibitory effect of YE4-CFE against DPP-4 was associated with the regulation of the expression of the genes related to the TNF and MAPK signaling pathways. The main active components of YE4-CFE were 19 kinds of small molecules with amolecular weight of less than 3 KDa, including adenine, acetylcholine, L-phenylalanine, etc. To the best of our knowledge, this is the first time examining the mechanism of CFE isolated from a LAB strain inhibiting the DPP-4 activity in Caco-2 cells. The findings of this study provide crucial information on the mechanism of the inhibitory effect of LAB strains against DPP-4 activity.

## Figures and Tables

**Table 1:** Compounds with a relative proportion higher than 1% in YE4-CFE with a molecular weight of less than 3 kDa by UHPLC-MS/MS.

Compounds	*m*/*z*	Class	Relative Content (%)
Adenine	119.0357	Imidazopyrimidines	10.573
Acetylcholine	146.118	Others	7.479
L-Phenylalanine	166.0867	Carboxylic acids and derivatives	7.177
Guanine	152.0572	Imidazopyrimidines	6.418
2-Hydroxycinnamic acid	165.0553	Cinnamic acids and derivatives	5.975
L-Tyrosine	182.0819	Carboxylic acids and derivatives	5.683
2′-Deoxyadenosine	252.1099	Purine nucleosides	4.321
Tetradecanedioic acid	257.1758	Fatty acyls	2.790
N-Benzylformamide	136.0762	Others	2.450
2-[2-(1-isobutylcyclohexyl)-1-methylethylidene]hydrazine-1-carboxamide	292.178	Others	2.301
Indole-3-acrylic acid	188.0713	Others	2.234
N-cyclooctylurea	171.1497	Others	1.982
Palmitic acid	274.2747	Fatty acyls	1.972
Uracil	113.0349	Diazines	1.628
Choline	104.1072	Organonitrogen compounds	1.521
3-Amino-4-methylpentanoic acid	130.087	Carboxylic acids and derivatives	1.501
Indole-3-lactic acid	206.0819	Indoles and derivatives	1.281
4-Hexylresorcinol	195.1235	Others	1.256
Azelaic acid	189.1129	Fatty acyls	1.054

## Data Availability

Not applicable.

## Supplementary Materials

The following are available online at https://www.mdpi.com/article/10.3390/foods11010080/s1. Figure S1: Identification of the *Lactobacillus plantarum* (*L. plantarum*) YE4 strain. (A) Colonial morphology of *L. plantarum* YE4. (B) Gram staining of *L. plantarum* YE4. (C) Sequence of the 16S rDNA gene of *L. plantarum* YE4. (D) Phylogenetic tree of *L. plantarum* YE4. (E) Test results of API 50CH kit for *L. plantarum* YE4. Table S1: Source information of lactic acid bacteria (LAB) strains. Table S2: Sequences of real-time quantitative PCR (qRT-PCR) primers used in this study. Table S3: Quality of the RNA-sequence data of each sample. CFE, treatment group after YE4-CFE interacted with Caco-2 cells; CTL, control group.
