# Supplementary material for: Inhibitory Effect and Potential Mechanism of Lactobacillus plantarum YE4 against Dipeptidyl Peptidase-4
Source: Foods. 2021 Dec 29;11(1):80. doi: 10.3390/foods11010080 (PMC8750294; doi:10.3390/foods11010080)
Supplement: Supplementary file 1 [file foods-11-00080-s001.zip › Supplementary Materials.pdf]

Table S1: Source information of lactic acid bacteria (LAB) strains.

System number	Strain number	Species	Source
2017SWU.B05.05015	M9	*Lactobacillus. plantarum*	Pickles isolate
2017SWU.B05.05011	M42	*Lactobacillus. plantarum*	Pickles isolate
2018SWU.B05.05007	26	*Lactobacillus. plantarum*	Yogurt isolate
2019SWU.B05.06001	127	*Lactobacillus. rhamnosus*	Yogurt isolate
2016SWU.B05.01002	L2	*Lactobacillus. casei*	Qula isolate
2018SWU.B05.08004	YE4	*Lactobacillus. plantarum*	Qula isolate
2014SWU.B05.02001	212	*Lactobacillus. fermentum*	Pickles isolate
2017SWU.B05.01001	R25	*Lactobacillus. casei*	Pickles isolate
2017SWU.B05.17001	P5	*Lactobacillus. pentosus*	Pickles isolate
2018SWU.B05.02019	H131	*Lactobacillus. fermentum*	Yogurt isolate
2018SWU.B05.05005	H130	*Lactobacillus. plantarum*	Yogurt isolate
2018SWU.B05.05004	H129	*Lactobacillus. plantarum*	Yogurt isolate

Table S2: Sequences of real-time quantitative PCR (qRT-PCR) primers used in this study.

Primer name	Accession number	Forward primer (5´to3´)	Reverse primer (5´to3´)
NFκBIA	NM_020529.3	CGCCCAAGCACCCGGATACAGC	TTCAGCCCCTTTGCACTCATAACG
CFLAR	NM_001351591.2	CTGTCCTTGTTCCTCGTCCC	ATAGCAACATCCCGGCACAA
ATF4	NM_001675.4	GTTTTGGATTGGTGGGGTGC	GTATTTGCCCCTCCCTGCTT
CREB3L3	NM_001271997.2	GACCTGCAACAGCATCACCT	TCTTCTCGGGTTGTGTCAGC
Tnfaip3	NM_001270507.2	GCCAAGAGAGATCACACCCC	TTCGTTTTCAGCGCCACAAG
JunB	NM_002229.3	CCTACCGGAGTCTCAAAGCG	TTGGTGTAAACGGGAGGTGG
IRAK1	NM_001025242.2	TTGAGAAGCACCCAGAGCAC	GCCAGCTTCTGGACCATCTT
FLNA	NM_001110556.2	ACAGTGTCAATCGGAGGTCAC	AAGCGCACACAGATGACGTA
STMN1	NM_001145454.3	ACTGGAACGTTTGCGAGAGA	ATCAATGCAGATTGGAGGCCC
HSPA1A	NM_005345.6	AGCTGGAGCAGGTGTGTAAC	CAGCAATCTTGGAAAGGCCC
HSPA1B	NM_005346.6	CTGGAGCAGGTGTGTAACCC	AGCAGCAAAGTCCTTGAGTCC
DUSP1	NM_004417.4	CAGAGCCCCATTACGACCTC	CCAGAGGAACTCGGGTGAAG
DDIT3	NM_004083.6	TTGCCTTTCTCCTTCGGGAC	CAGTCAGCCAAGCCAGAGAA
MYC	NM_001354870.1	TACACTAACATCCCACGCTCTG	TTCTCTGCTGCTCCTCCGTA
NR4A1	NM_001202233.2	TTCCTGGTGTAAGCTTTGGTATGG	ACTCTGGATGGACCAACAGG
GAPDH	NM_001357943.2	GGGCTGCTTTTAACTCTGGT	TGGCAGGTTTTTCTAGACGG
DPP-4	XM_003820939.3	CGGAGTCCTGGGTTTCAGTT	CCTCCAACCTCACGTGGAC
PpiA	XM_007981418.2	TGCTGACTGTGGACAACTCG	TGCAGCGAGAGCACAAAGAT

Table S3: Quality of the RNA-sequence data of each sample. CFE, treatment group after YE4-CFE interacted with Caco-2 cells; CTL, control group.

Sample	Raw reads(bp)	Clean reads(bp)	Q20 (%)	Q30 (%)
CFE-1	8495256900	8348818788	97.79%	93.68%
CFE-2	7162500900	7057336417	97.73%	93.55%
CFE-3	7621356900	7491625117	97.58%	93.22%
CTL-1	6556791300	6468598771	97.53%	93.16%
CTL-2	7233822000	7134179679	97.71%	93.48%
CTL-3	7041612300	6924493214	97.55%	93.18%)

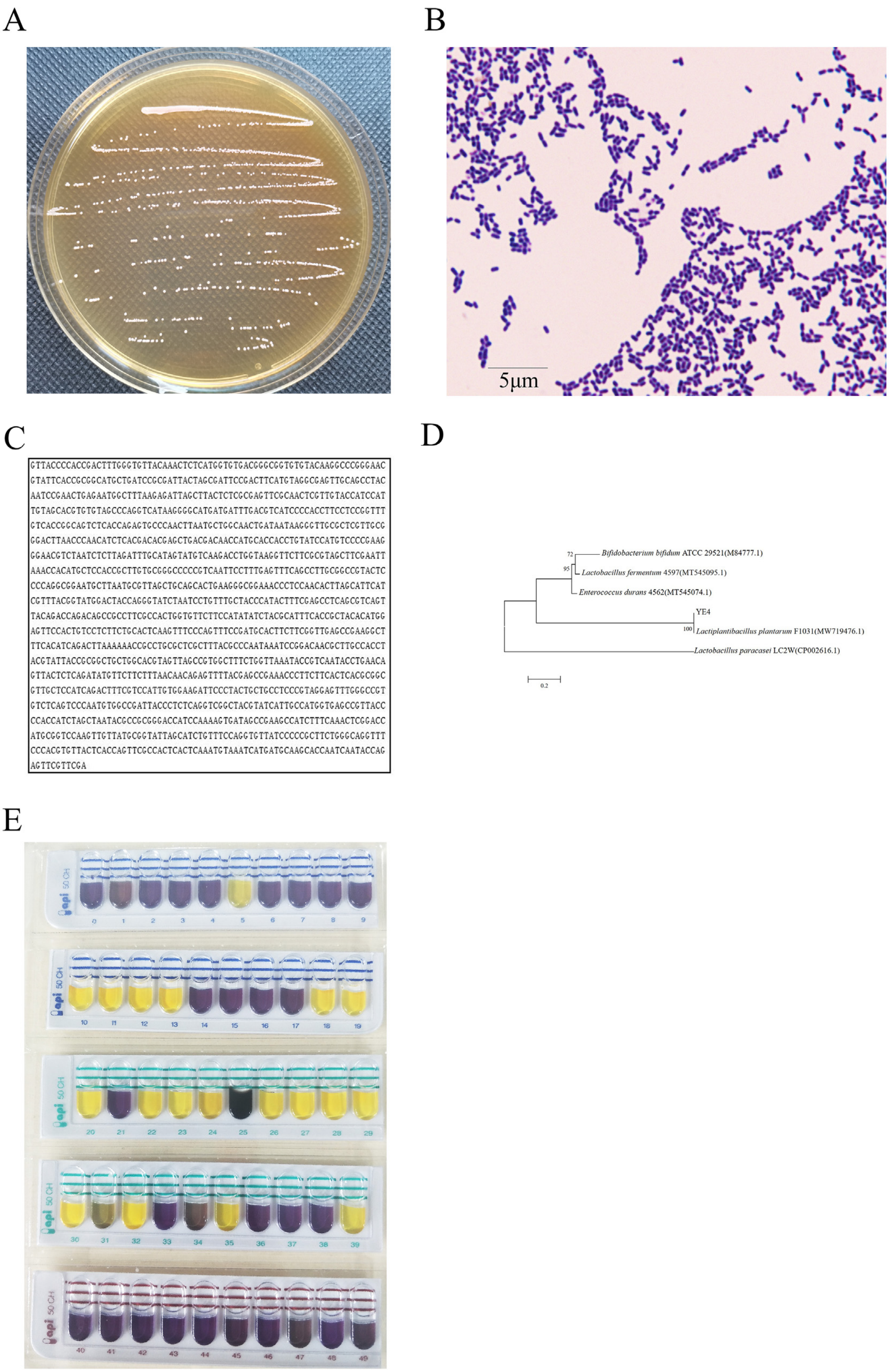

Figure S1: Identification of the *Lactobacillus plantarum* (*L. plantarum*) YE4 strain. (A) Colonial morphology of *L. plantarum* YE4. (B) Gram staining of *L. plantarum* YE4. (C) Sequence of the

16S rDNA gene of *L. plantarum* YE4. (D) Phylogenetic tree of *L. plantarum* YE4. (E) Test results of API 50CH kit for *L. plantarum* YE4.
